# A Review of the Efficacy of Influenza Vaccination in Autoimmune Disease Patients

**DOI:** 10.7759/cureus.15016

**Published:** 2021-05-13

**Authors:** Mandi Abdelahad, Elizabeth Ta, Marc M Kesselman, Michelle Demory Beckler

**Affiliations:** 1 College of Osteopathic Medicine, Nova Southeastern University Dr. Kiran C. Patel College of Osteopathic Medicine, Fort Lauderdale, USA; 2 Rheumatology, Kiran C. Patel College of Osteopathic Medicine, Nova Southeastern University, Davie, USA; 3 Microbiology and Immunology, Nova Southeastern University Dr. Kiran C. Patel College of Allopathic Medicine, Fort Lauderdale, USA

**Keywords:** systemic autoimmune disease, influenza vaccine, systemic lupus erythematosis, sjogren's, rheumatoid arthritis, inflammatory bowel disease

## Abstract

Patients suffering from autoimmune diseases appear to be at greater risk for developing infections with the influenza virus compared to healthy controls due to their immunosuppressive treatment, suggesting the importance of vaccination. Within this literature review, we highlight the importance, efficacy, and safety of influenza vaccination in individuals with autoimmune diseases, including systemic lupus erythematosus (SLE), Sjogren syndrome (SS), rheumatoid arthritis (RA), and inflammatory bowel disease (IBD) in both vaccinated and unvaccinated individuals. Overall, vaccination is generally well tolerated by SLE patients and the literature recommends the inactivated influenza vaccine to SLE patients according to the recommendations and schedules for the general population and annually against seasonal influenza viruses. While the data are still unclear in patients with SS, there does seem to be a general consensus to vaccinate these individuals to prevent harmful risks of influenza disease. In patients with RA and IBD, vaccination efficacy with the inactivated influenza vaccine should be determined on a case-by-case basis, taking patient therapy into account. In light of the current pandemic and global coronavirus disease 2019 (COVID-19) crisis, it is crucial to emphasize the safety and immunogenicity of influenza vaccination in vulnerable individuals suffering from autoimmune diseases. Public health measures are recommended to protect these individuals with vaccinations, keeping in mind the possibility of the multiple COVID-19 vaccines that are currently available.

## Introduction and background

Introduction

Autoimmune diseases such as rheumatoid arthritis (RA), systemic lupus erythematosus (SLE), and multiple sclerosis (MS) have been shown to be associated with dysregulated immune system activity [1]. This likely results from an individual’s own immune system attacking its own healthy cells. Immune system recognition of auto-antigens can lead to tissue, organ, and/or systemic damage. To date, the exact underlying mechanisms of many autoimmune diseases have yet to be fully elucidated. However, modifications in the selection, regulation, and/or death of T-cells or B-cells, and/or abnormal responses to antigens appear to be associated with the onset and progression of many autoimmune diseases [1]. The production of autoantibodies may also result in tissue injury and inflammation by the formation of immune complexes (as in SLE), cytolysis (as in RA), or epitope spreading (as in Sjogren’s), and interference with cellular physiology. While immune mechanisms play a vital part in the pathogenesis of autoimmune diseases, onset and progression are thought to be multi-factorial [2]. Treatment of autoimmune diseases most commonly involves immune suppression using anti-inflammatory agents, immunosuppressants, steroids, and other more targeted biologic agents. Due to the immunosuppressive nature of these treatment strategies, there may be an increased risk of infection and an associated increased risk in morbidity and mortality in patients with autoimmune diseases [2-4].

Background

Influenza viruses A and B strains are highly transmissible human pathogens. Recent studies show that during a typical influenza season, 5% to 20% of the United States population become ill with the virus, including more than 200,000 individuals who are hospitalized and approximately 36,000 individuals who die as a result of the virus or complications associated with the viral infection [3]. Globally, there are three to five million cases and up to 500,000 deaths [4]. Studies are currently underway evaluating the use of trivalent and quadrivalent inactivated influenza vaccines (IIVs) in autoimmune disease patients and are thought to be the most efficient strategy to reduce the mortality and morbidity associated with influenza. Two main types of influenza vaccines are currently licensed in the United States: trivalent or quadrivalent IIV and live attenuated influenza vaccine (LAIV). Both vaccines contain the predicted antigenic variants of influenza A/H3N2, A/H1N1, and B viruses; the main difference is in their triggered response [4]. TIV contains purified hemagglutinin protein (HA) and elicits a higher serum IgG antibody response than LAIV in healthy individuals. LAIV contains a weakened virus and is administered as a nasal spray, which elicits a better IgA mucosal response than IIV in healthy individuals, helping to prevent an infection prior to viral replication [4]. LAIV is contraindicated in patients with autoimmune disease receiving biologic medications because of a risk that the vaccine could cause the flu or comorbid disease flare, whereas IIV is recommended for these patient populations. In this review, we highlight studies involving the use of influenza vaccination in patients who are immunosuppressed and present with an autoimmune disease.

## Review

Systemic lupus erythematosus (SLE) presentation, pathophysiology, and treatment

SLE is a chronic autoimmune disorder, which commonly impacts teenagers and adults, aged 15 to 44 years [5]. These patients typically present with fever, fatigue, skin rashes, arthritis, and kidney complications. While the underlying mechanism of disease activity has yet to be fully elucidated, a combination of genetic susceptibility, environmental factors, and the development of pathogenic auto-antibodies is likely involved [6]. These antibodies include anti-nuclear antibodies that can be to double-stranded DNA (dsDNA), histones, other extractable nuclear antigens (ENAs) such as the Smith antigen (Sm), SSA/Ro, SSB/La, RNP, and C1q [6]. Ultimately, the underlying SLE immune mechanisms and hypersensitivity reactions lead to destructive inflammatory responses and tissue injury [7]. 

Patients with SLE present with symptoms that are cyclical, which include periods of remission and flares. Treatment commonly focuses on immune suppression. Milder SLE cases are often treated with antimalarials, such as hydroxychloroquine, in combination with nonsteroidal anti-inflammatory drugs (NSAIDs) or corticosteroids and with disease progression, azathioprine, mycophenolate, methotrexate, or belimumab can be used [8]. In 2011, the Food and Drug Administration (FDA) approved belimumab, a human monoclonal anti-BAFF (B-cell activating factor) antibody, for the treatment of active SLE and flares in adult patients. This is the first new drug approved for SLE since 1955 [8]. BAFF is important for the activation of autoreactive B-cells in SLE. A common side effect of many immunosuppressants includes immunodeficiency, increasing a patient’s susceptibility to various infections and secondary diseases, such as bronchitis, cystitis, and pharyngitis [6,8].

SLE and the use of influenza vaccination

SLE patients are at a greater risk for developing influenza infections as compared to their healthy counterparts due to their immunosuppressed condition [9,10]. Many studies have demonstrated that influenza vaccination with the inactivated vaccine in SLE patients results in lower hospitalizations due to influenza infection or complications of the infection, which included pneumonia and acute bronchitis, as compared with unvaccinated SLE patients [10,11]. In addition, SLE patients who underwent vaccination with the trivalent influenza vaccine had no immunization-related disease flares and were less likely to be predisposed to death as compared to their non-vaccinated SLE counterparts [10,11]. There was no evidence of worsening of SLE disease after immunization [10,11]. Research concluded that these patients run a greater risk of disease exacerbation as a result of viral or bacterial infections if not vaccinated with the inactivated A/H1N1 vaccine [12]. Thus, the general recommendation is yearly influenza vaccination in SLE patients, which was shown to be safe and generated a good humoral response in these patients [10-12].

Despite the overwhelming amount of literature regarding positive outcomes in vaccinated SLE patients as compared to healthy controls (HCs), a few studies have shown SLE patients exhibit lower immune response rates as compared to HCs [13]. For example, although seroconversion rates were lower in SLE patients on azathioprine compared to HCs, SLE patients had the same rate of seroprotection, suggesting vaccination does provide SLE patients benefit [13]. The use of azathioprine in SLE patients resulted in four-fold fewer antibody titers compared to control groups, suggesting a decreased immune response to influenza vaccination in these subjects [5,13,14]. Prednisone was also reported to lower the immune response to the influenza vaccine using the same criteria [5]. Vaccinated SLE patients on immunosuppressive treatment had lower seroprotection and seroconversion rates compared to HCs. Without immunosuppressive treatment, however, there was no significant difference in seroprotection rates between SLE patients and HCs [15]. This study concluded SLE patients receiving azathioprine and prednisone had lower seroprotection rates in SLE patients than HCs [15].

Administration of a single dose of the inactivated influenza vaccine was impaired in patients receiving immunosuppressive drugs such as mycophenolate mofetil, azathioprine, cyclophosphamide, methotrexate, or prednisone [15,16]. Administering a second dose of the inactivated influenza vaccine was shown to increase vaccine immunogenicity and did not result in increased disease flare, even in subjects with active SLE [6]. Certain immunosuppressive drugs, such as hydroxychloroquine, do not appear to interfere with a second dose of the influenza vaccine and do not decrease the seroprotection rates of SLE patients when vaccinated as compared to HCs [15,16]. 

Overall, there are many studies indicating safe and effective use of the inactivated influenza vaccine in SLE patients, but providers should keep in mind the immunosuppressive treatment regime of the SLE patient when recommending one or two doses of the inactivated vaccine [5,15,16].

Sjogren Syndrome (SS) presentation, pathophysiology, and treatment

SS is a systemic autoimmune disorder that can occur at any age but typically develops gradually between the ages of 30 and 50 years, more commonly in women than men. In this condition, there appear to be autoantigens within the lacrimal and salivary exocrine glands [17,18]. The resulting immune reactions, including lymphocyte infiltration and the presence of inflammatory cytokines, primarily impair the ability of these glands to secrete fluids, such as tears and saliva, leading to the main sicca symptoms that include keratoconjunctivitis sicca (dry eyes) and xerostomia (dry mouth) [18]. The immune system can also attack other organs and tissues in some cases, leading to extraglandular involvement that may result in arthritis, rash, interstitial lung disease (ILD), neurological issues, primary biliary cirrhosis/cholangitis, renal tubular acidosis, or lymphoma. In extreme cases, patients with SS may develop B-cell lymphomas at a higher rate than the general population [17,18]. Similar to SLE, the underlying mechanism of disease activity in SS has yet to be fully elucidated but is likely attributable to a combination of genetic predisposition and environmental factors. A key contributing factor in SS symptom development is the presence of abnormal B- and T-cell responses to the presence of the auto-antibodies SS-related antigen A and SS-related antigen B (anti-Ro/SSA and anti-La/SSB autoantibodies, respectively), which are detected in up to 90% and 60% of patients with SS, respectively [17].

SS treatment can include drugs such as symptomatic relief of dry eyes with hydrating or immunosuppressant eye drops, therapies to increase saliva formation, and encouraging drinking water to help to combat the symptoms of dry mouth. In some patients, hydroxychloroquine and methotrexate have both been beneficial in decreasing systemic pain and salivary gland swelling [19]. Still, both drugs may increase a patient’s susceptibility to various infections.

SS and the use of influenza vaccination

Data evaluating the efficacy and safety of the influenza vaccine in SS patients remain scarce and findings regarding safety have been mixed. While some studies report the inactivated vaccine to be well-tolerated in patients with SS, other studies cautioned the use of the vaccine due to the resultant expression of SS-related auto-antibody profiles in SS patients after vaccination and mainly in anti-Ro (SSA)/La(SS-B) [20]. Meanwhile, no studies to date have reported any serious adverse clinical presentations after vaccination with the inactivated vaccine. One cross-sectional study examined the efficacy and safety of the influenza vaccine in three groups of patients with SLE, RA, and SS as compared to a non-vaccinated control group of those same diseases by using serological tests and hemagglutination inhibition assays with influenza virus antigens [21]. Patients with SS in the vaccinated group had fewer occurrences of influenza and lower total viral infections compared to the unvaccinated group. In addition, this study indicated the vaccine was well-tolerated in all patients with SS and there were no cases of disease exacerbation [21].

Influenza vaccination can exacerbate the auto-antibody profile of SS, without altering the clinical disease course, suggesting that this antibody production could be a result of molecular mimicry [17,20]. Vaccinated SS patients with the inactivated vaccine have similar rates of seroconversion and seroprotection compared to HCs [17] and develop higher anti-H1N1 IgG antibodies with greater avidity than HCs [20]. Yet, vaccinated SS patients exhibit important long-term (one year) changes, including a significant increase in the levels of anti-Ro/SSa and anti-La/SSB, which has been shown to be associated with an increase in B-cell activation in SS patients after influenza vaccination [17,20]. This increase in B-cell activation was not seen in SS patients treated with hydroxychloroquine [20]. Still, no significant differences in clinical presentations of fever, fatigue, myalgia, or arthralgia were observed between SS patients and HCs subsequent to vaccination [20].

Overall, the data regarding immunogenicity in SS patients remain limited. While there seems to be a general agreement with regards to vaccination in patients with SS to prevent more harmful risks of disease, the efficacy and safety of the inactivated influenza vaccine in SS patients require further study [17,20,21].

Rheumatoid arthritis (RA) presentation, pathophysiology, and treatment

RA is the most common form of inflammatory arthritis, affecting approximately 1% of the population [22,23]. RA mainly affects the joints, causing chronic inflammation and articular destruction, sometimes leading to chronic pain, architectural and radiographic bony destruction, and extra-articular disease manifestations to organs such as the lungs, heart, and eyes [24]. Genetic factors, such as rheumatoid factor (RF) or HLA-DR4 and PTPN22 alleles have been strongly associated with RA [24]. These RA-associated alleles appear to present different peptides, such as citrullinated proteins, which are arthritogenic antigens [24]. As a result, anti-citrullinated peptide antibodies (ACPAs) are found in approximately 80% to 90% of RA patients [24]. In addition, environmental factors, such as smoking, may play a role in RA onset and progression by altering the mucosal surfaces of various organs in RA patients [24], leading to translational modification of peptides, specifically the conversion of arginine to citrulline within the airway and the production of ACPAs [22,24].

RA can be effectively treated and managed with various therapies, such as NSAIDs, corticosteroids, biologic, and nonbiologic disease-modifying anti-rheumatic drugs (DMARDs) [25-27]. Rituximab and anti-tumor necrosis factor (TNF) alpha drugs are biological therapies used in RA patients [28,29]. Rituximab depletes B-cells for six to nine months, while anti-TNF alpha drugs inhibit effects of the cytokine TNF alpha [28,29]. Here, we focus on biologic and non-biologic DMARDs and their role in the efficacy of influenza vaccination in RA patients. 

RA and the use of influenza vaccination

Patients with RA undergoing immunomodulatory treatments with biologic and non-biologic DMARDs are considered immunosuppressed, putting these patients at an increased risk for developing infections, especially involving the respiratory tract [23,28]. Thus, it is recommended by the American College of Rheumatology (ACR) that all RA patients receive a yearly influenza vaccination, as the vaccine has been associated with a reduced risk of hospitalization for septicemia, bacteremia, and/or viremia [23,28]. Still, the number of RA patients receiving vaccinations remains low compared to the general population, potentially attributable to concerns regarding the efficacy and safety of vaccines in patients on immunosuppressive therapies [30].

Abatacept is a soluble fusion protein that selectively modulates the CD28:CD80/86 co-stimulatory signal that is required for T-cell activation and is recommended for RA patients naïve to biologic medications as well as those with an inadequate TNF-alpha blocker response [30]. When RA patients receiving subcutaneous abatacept with adjunctive DMARDs were vaccinated with the 2011-2012 trivalent seasonal influenza vaccine, 81% of RA patients on abatacept reached protective antibody levels of titers greater than 1:40 to two or more of three antigens, 28 days post-vaccination [30]. These results indicate that trivalent influenza vaccination appears to be effective in RA patients receiving subcutaneous abatacept and adjunctive DMARDs [30,31]. 

One of the most common biologic treatments for RA is TNF-alpha blockers, including etanercept and infliximab [32,33]. The data remain mixed as to whether these drugs impair vaccine response in RA patients [33]. When RA patients receiving DMARDs, including TNF alpha-blockers, were vaccinated with the split-virion inactivated influenza vaccine, the researchers found significant increases in geometric mean titers (GMTs) of hemagglutination inhibiting (HI) antibodies for each antigen among subjects [33]. In addition, the percentage of humoral responses, measured by the GMT of HI antibodies to the inactivated influenza vaccine, was not affected by any DMARD, including methotrexate, infliximab, and etanercept [33]. Meanwhile, when the trivalent influenza vaccine was administered to RA patients, patients treated with only methotrexate had higher antibody titers specific for influenza antigens as compared to patients that were treated with TNF-alpha blockers with or without methotrexate. This result suggests TNF blockers impair serological response to the trivalent influenza vaccine [32]. Overall, it appears that either the type of vaccine used or concomitant drug therapies can affect vaccine efficacy in RA patients treated with TNF-alpha inhibitors [32,33].

Additional studies have evaluated the humoral responses of RA patients treated with immunosuppressive therapies after trivalent influenza subunit vaccine administration and found that the GMT of antibodies against the A/H3N2 and B strains prior to vaccination was higher in HCs, likely due to influenza vaccination during the previous season [28]. In addition, 28 days following vaccination, GMT increased in HCs and in RA patients treated with methotrexate [28]. Contrastingly, in patients treated with rituximab, a CD20 monoclonal antibody that depletes CD20+ B-cells in circulation, the timing of treatment played a major role in the response to vaccination [28,31]. Patients treated with rituximab six to 10 months prior to vaccination showed increases in GMT and CD19+ B-cell numbers after vaccination [28]. Meanwhile, there was no increase in either influenza GMT or CD19+ B-cell numbers in patients treated with rituximab four to eight weeks prior to vaccination [28]. HCs achieved greater seroprotection for the A/H1N1 strain compared to the rituximab group treated four to eight weeks prior to vaccination [28]. While patients treated with rituximab six to 10 months prior to vaccination did exhibit better humoral responses to influenza vaccination compared to those patients that were treated with rituximab four to eight weeks prior to vaccination, the increase in GMT of the rituximab patients was still lower than the GMT of HCs or methotrexate-treated patients [28]. A separate study found similar results of impaired humoral response to trivalent influenza vaccination in RA patients receiving rituximab [31]. These results suggest that the humoral response to influenza vaccination is both decreased and transient in RA patients undergoing rituximab therapy, with the timing of rituximab treatment playing a key role [31,34].

Though the immunosuppressive effect of methotrexate may be beneficial to RA patients, it may also compromise vaccine efficacy [34]. Of note, current studies suggest there may be a benefit to RA patients who temporarily discontinue methotrexate while receiving the influenza vaccine [34]. In RA patients who held methotrexate for two weeks before and two weeks after vaccination with trivalent influenza vaccine, patients achieved significantly higher vaccine responses when compared to patients who continued methotrexate [34]. Similarly, withholding methotrexate for two weeks after vaccination with quadrivalent influenza vaccine resulted in more than a four-fold increase in influenza antibody titers when compared to patients who continued methotrexate [34].

Patients with RA receiving methotrexate or abatacept therapy appear to have a higher antibody response to trivalent influenza vaccination [28,30,32]. RA patients on methotrexate may have a greater immune response to influenza vaccine by holding therapy for two to four weeks after vaccination with trivalent or quadrivalent influenza vaccine [34]. RA patients treated with rituximab showed a decreased level of humoral immune response to the trivalent subunit influenza vaccine, likely due to the drug’s B-cell inhibition, but a similar cell-mediated response to the vaccine compared to patients receiving methotrexate [23,28,35]. The humoral response was slightly improved in the RA patients who received the influenza vaccination six to 10 months after rituximab treatment compared to patients who received the vaccine four to six weeks after rituximab treatment [28]. Patients who had been previously vaccinated with influenza were able to reach higher anti-influenza titers to the A/H1N1 strain [28]. TNF blockers cause the most impaired serological response to the trivalent influenza vaccine [32]. Still, some patients were able to mount a good humoral response to inactivated influenza vaccination despite DMARD and/or anti-TNF therapy [31].

Overall, the literature suggests the immune response to influenza vaccination in RA patients varies and may or may not be limited by treatment with immunosuppressive medications. Therefore, vaccine efficacy should be determined on a case-by-case basis, taking patient therapy into account, with further investigation warranted.

Inflammatory bowel disease (IBD) presentation, pathophysiology, and treatment

IBD, consisting of Crohn’s disease (CD) and ulcerative colitis (UC), results in inflammation of the gastrointestinal tract (GI) and impairs the GI tract from absorption, digestion, and elimination [36-38]. The most common manifestations are persistent diarrhea, abdominal pain, rectal bleeding, weight loss, and fatigue [36,38]. Both CD and UC are autoimmune diseases affecting the GI tract with different underlying pathogenic mechanisms, but both involve multifactorial triggers, including environmental, microbial, and genetic [37].

IBD pathogenesis has been linked to mucosal immunity, in which autoreactive immune responses occur with the mucosa [37]. Studies suggest that the pathogenesis of CD is driven by a Th1 response, while UC is associated with a Th2 response, with both resulting in chronic inflammation of the GI mucosa [37]. Accordingly, IBD can be managed with anti-inflammatory drugs such as corticosteroids and aminosalicylates (mesalamine) as the first-line treatment [39-41].  Two classes of biologics used to control the symptoms of IBD include anti-TNF medications that block the protein and anti-integrin therapy that inhibits lymphocytes from binding to the lining of the gastrointestinal tract.

IBD and the use of influenza vaccination

Similar to other autoimmune diseases, there is a recommendation for yearly influenza vaccination in IBD patients, yet the vaccination rates for IBD patients remain low [39-44]. In a cohort of 255 IBD adults who received the inactivated influenza vaccine between 2009-2010 and 2010-2011, researchers found high seroprotection rates in IBD patients not receiving any treatment, as well as in IBD patients with immunosuppressive treatment not including anti-TNF therapy after vaccination [40]. The persistence of seroprotection three weeks and six months post vaccination was lower in IBD patients on TNF-alpha blockers with or without additional therapies. This study showed similar results after the second year of vaccination compared to control groups without TNF-alpha blockers [40]. These results suggest that TNF-alpha inhibitors impair serological and immune response in IBD patients vaccinated with the inactivated influenza vaccine [40].

In a study including 146 children and young adults with IBD, researchers evaluated their immune response to the trivalent influenza vaccine [45,46]. The proportion of patients seroprotected against influenza A strains, H1N1 and H3N2, were similar between HCs, non-immunosuppressed, and immunosuppressed groups, suggesting that influenza vaccination was protective regardless of immunosuppression status [46]. Influenza B strain was less immunogenic across all groups of healthy, non-immunosuppressed, and immunosuppressed patients. However, those receiving TNF inhibitors had a significantly lower protection rate against strain B compared to the non-immunosuppressed group (14% and 39%, respectively) [46]. Surprisingly, although tacrolimus and TNF-alpha inhibitors are both immunosuppressive therapies that affect T-cells, patients taking tacrolimus had a similar proportion of seroprotection as HCs [46]. Similarly, IBD patients on infliximab achieved seroprotection to the trivalent influenza vaccine, albeit at lower numbers than controls [41]. However, the vaccine timing relative to infliximab infusion did not decrease effectiveness, and the vaccine is considered safe and is recommended for use in this patient cohort [41].

However, IBD patients on immunosuppressive therapy that were vaccinated with non-adjuvant influenza A monovalent vaccine had decreased seroprotection compared to non-immunosuppressed treated patients [45]. Patients on combination immunosuppression (defined as two or more of prednisone, thiopurine, methotrexate, or a biological drug) had an even lower rate of seroprotection compared to subjects, not on combination immunosuppressive treatment [45]. These results suggest that IBD patients vaccinated with the inactivated H1N1 influenza vaccine had low rates of seroprotection, especially if they were immunosuppressed [45].

Influenza vaccination, especially with A strains, appears to be protective against influenza in IBD patients and should be recommended, despite the use of immunosuppressive agents [46]. Patients with IBD receiving anti-TNF therapy, such as infliximab, may experience impaired serological response to inactivated influenza vaccine, particularly B strains [40,41,45,46]. Despite decreased seroprotection, the inactivated vaccine is still considered safe and recommended for IBD patients on anti-TNF therapy at any point during treatment [41]. Patients on combination immunosuppressive medication may have a low rate of seroprotection compared to non-immunosuppressed patients [45]. Due to varying vaccine effectiveness across various IBD treatment approaches, vaccination should be determined on a case-by-case basis.

## Conclusions

Studies have shown that the inactivated influenza vaccine is safe and efficacious in patients with SLE, RA, and IBD. It reduces the risk of infections, hospitalizations, and exacerbation of the autoimmune disease. On the other hand, there have been studies with mixed results establishing the safety of the influenza vaccine in SS patients. While some studies recommend vaccination in patients with SS, others caution about vaccine use due to the increased auto-antibody profiles in SS patients. All in all, these recommendations may be determined on a case-by-case basis depending on each patient’s treatment regime and preference towards vaccination. It is important to emphasize the safety of vaccinating vulnerable individuals suffering from autoimmune diseases on immunosuppressive therapy with vaccines that have been proven safe and effective, particularly in light of the current COVID-19 pandemic. Public health measures are warranted to protect these individuals with routine vaccinations, keeping in mind the possibility of the multiple COVID-19 vaccines that are currently available.
